# Towards the Use of Unmanned Aerial Systems for Providing Sustainable Services in Smart Cities

**DOI:** 10.3390/s18010064

**Published:** 2017-12-27

**Authors:** Enrique Moguel, José M. Conejero, Fernando Sánchez-Figueroa, Juan Hernández, Juan C. Preciado, Roberto Rodríguez-Echeverría

**Affiliations:** Quercus Software Engineering Group, INTIA (Instituto de Investigación en Tecnologías Aplicadas de Extremadura), University of Extremadura, Cáceres, Spain; chemacm@unex.es (J.M.C.); fernando@unex.es (F.S.-F.); juanher@unex.es (J.H.); jcpreciado@unex.es (J.C.P.); rre@unex.es (R.R.-E.)

**Keywords:** UAS, technical sustainability, drones

## Abstract

Sustainability is at the heart of many application fields where the use of Unmanned Aerial Systems (UAS) is becoming more and more important (e.g., agriculture, fire detection and prediction, environmental surveillance, mapping, etc.). However, their usage and evolution are highly conditioned by the specific application field they are designed for, and thus, they cannot be easily reused among different application fields. From this point of view, being that they are not multipurpose, we can say that they are not fully sustainable. Bearing this in mind, the objective of this paper is two-fold: on the one hand, to identify the whole set of features that must be provided by a UAS to be considered sustainable and to show that there is no UAS satisfying all these features; on the other hand, to present an open and sustainable UAS architecture that may be used to build UAS on demand to provide the features needed in each application field. Since this architecture is mainly based on software and hardware adaptability, it contributes to the technical sustainability of cities.

## 1. Introduction

Sustainability has become one of the priority of many public administrations’ and governments’ roadmaps. Indeed, sustainability goals are present in many political campaigns, governmental programs and global meetings. Such interest in sustainability spreads across countries and at a national level is being promoted with dedicated funds for innovation and development in a wide range of areas. A clear example of these areas where sustainability is being strongly fostered is smart cities [1,2,3], where the appearance of new technologies and the reduction of their prices are making it possible to provide new services to, among others, reduce the consumption of natural resources or the costs associated with traditional services provided by local administrations.

In that sense, one of the common technologies used in smart cities is a cyber-physical system [4,5] that allows the acquisition of data from the city with the aim of being able to take important and corresponding decisions [6,7,8] (e.g., automatically adapting water irrigation in terms of weather conditions). However, the acquisition and installation of this wide network of sensors has important costs for the cities that, sometimes, make this solution unsustainable for the smallest ones. This is where **UAS** (Unmanned Aerial Systems, commonly known as **drones**) come to the scene [9] since they may include a wide variety of sensors that allow the city not only to measure (with a single device) many physical magnitudes, but also to do so dynamically, at different locations [10,11], for a reduced price compared to the alternatives more commonly used for the same purpose. Based on these advantages, UAS are being fostered in many domains, many of them related to smart cities, especially those where mapping and remote sensing techniques must be applied [12,13,14] or where autonomous navigation in GNSS-denied environments is required [15,16,17,18,19]. As examples, we can cite agriculture [20], airborne surveillance [21], aerial photography [22], detection of gas leaks [23,24], detection and prediction of fires [25], environment surveillance [26], archeology [27], monitoring of photovoltaic systems [28], supervision of structures and buildings [29] or inspection of power lines [30]. UAS provide important benefits in these domains mainly due to their energetic efficiency, their reduced carbon footprint and, as previously mentioned, their decreasing cost, when compared to other alternatives [31,32].

The appearance of all these varying fields implies also the appearance of several features in UAS. Just to cite a few, some of them require storage capabilities; others require communicating with a third party for acquiring or providing data during flight; others require programming the route before flying and re-programming it in real time; and there are others that require some level of computational capabilities on board for pre-processing images or running algorithms to avoid obstacles or make decisions during the flight. Being aware of the demand for these new features, on the one hand, many companies have incorporated them into the UAS that they commercialize (e.g., DJI (DJI: http://www.dji.com) or Parrot (Parrot: https://www.parrot.com/us/)), and on the other hand, do-it-yourself UAS are being built ad-hoc to be applied in these particular fields. However, these UAS either have a proprietary architecture that makes their extensibility difficult or they are built for each application field and do not provide all the features required in others, respectively. Thus, again, the solution may be unsustainable for many cities due to the need for acquiring different UAS for each application or purpose.

Precisely, the main goal of this work is to present an open and sustainable UAS architecture that may be used to build UAS on demand to provide the features needed in each application field. Note that this UAS architecture could solve this problem in a sustainable way since it might be used in all these application fields with clear benefits for the cities providing services with it: do more with less. With a smaller fleet of UAS, they can cover a wider set of application fields, saving the costs of acquiring new devices each time or saving costs in maintenance. This is aligned with the ideas about sustainability in smart cities presented in [9], where both efficiency in infrastructure and services and reduced costs are mandatory. Additionally, since this architecture is mainly based on software and hardware adaptability, it will contribute to technical sustainability in cities, defined by [33] as the long-time usage of software systems and their adequate evolution over time.

The rest of paper is structured as follows. Related works are introduced in Section 2. Section 3 presents the background regarding the main areas related to this work. Section 4 briefly describes the whole process performed in this work to evaluate the need for a sustainable UAS. Section 5 and Section 6 present the open multipurpose architecture and a UAS built based on it, respectively. Finally, Section 7 concludes the paper.

## 2. Related Works

UAS are currently being used in many areas related to sustainability. Some representative examples of these works where UAS are being used are given next. In [34], the authors propose the use of UAS to monitor the air quality (environmental dimension) in order to reduce the costs of using expensive satellites or balloons (economic dimension). Similarly, the work in [35] measures nuclear radiation indexes in places where there is a danger for people due to nuclear catastrophes (environmental dimension). As the authors claim in the work, based on the use of UAS, they could benefit from important reductions in costs and time in these tasks. In [36,37], the authors make use of UAS with the aim of supporting medical services in tasks of rescuing people in environmental disasters (social and environmental dimensions) or similar. In [38], the authors use UAS to take orthophotos of vast extensions with geospatial purposes (environmental dimension). Some electric companies are also starting to use UAS to monitor electric towers [39], reducing the costs of these operations (economic dimensions) and reducing the risk for their employees (social dimension).

However, although all these works focus on the application of UAS with sustainable purposes, just a few works have dealt with making a sustainable use of UAS. For instance, the works in [40] or [41] have proposed some techniques to optimize the consumption of energy by the UAS so that the routes may be adapted according to this consumption. Similarly, there are some approaches to make the UAS self-sufficient in terms of being able to produce the resources that they need to operate (e.g., auto-charging the batteries by means of photovoltaic devices [42]). Other examples are the approaches that propose the use of algorithms to allow collaborative work among UAS so that their tasks may be coordinated in order to save time and costs [43,44,45]. Nevertheless, to the best of our knowledge, there is a lack of approaches that propose the use of a generic and multi-purpose UAS architecture that may be used in all these domains and with all these purposes, like the approach presented in this paper. Note that the architecture presented here relies on the use of adaptive techniques not only for the UAS hardware, but also for its software.

## 3. Background

In order to make the paper self-contained, this section provides the background regarding the two main areas related to our work: (i) sustainability in software development and (ii) UAS.

### 3.1. Sustainability in Software Development

In the last few years, the software engineering community has made an important effort to introduce sustainability as a primary focus in software development [46,47,48,49]. Therefore, it is not surprising that several definitions of sustainability have been recently provided, some of them collected in [50], where for example, software sustainability is defined as a composite, non-functional requirement, which is a measure of a system’s extensibility, interoperability, maintainability, portability, reusability, scalability and usability [50]. The Software Sustainability Institute claims that sustainability means that the software you use today will be available (and continue to be improved and supported) in the future [50]. Naumann et al. distinguish between sustainable software and sustainable development. While sustainable software is defined as software, whose direct and indirect negative impacts on the economy, society, human beings and the environment that result from development, deployment and usage of the software are minimal and/or which has a positive effect on sustainable development, sustainable software development is defined as the art of developing sustainable software with a sustainable software engineering process so that negative and positive impacts result in and/or are expected to result from the software product over its whole life cycle and are continuously assessed, documented and used for further optimization of the software [51].

There are several categorizations of sustainability. The United Nations defined a set of ten themes ranging from economic to social aspects of sustainability [52]. Goodlan also provided a categorization for general sustainability based on four different dimensions: individual, social, economic and environment [53]. The latter three were also defined as themes by the United Nations. However, as was claimed in [33], an additional category related to technology is needed in order to consider sustainability in software systems such that they added one dimension to those considered by Goodlan, the technical dimension. These five dimensions are defined as follows:

- Individual sustainability refers to private goods and individual human capital.
- Social sustainability relates to societal communities (mainly based on solidarity).
- Economical sustainability refers to assets, capital and, in general, added value achieved by the improvement of sustainability in a particular context.
- Environmental sustainability includes those activities performed to improve human welfare by protecting natural resources.
- Technical sustainability relates to the long-time usage of software systems and their adequate evolution over time.

The usage of UAS to perform complex actions in the cities has a significant impact mainly on the economic (due to the reduction in costs) and environmental (based on the many applications related to this area) dimensions. However, as will be shown in this work, the approach presented here also impacts technical sustainability, since the proposed architecture promotes extensibility and reusability, improving, thus, the long-term usage of the system and UAS built.

### 3.2. Unmanned Aerial Systems

The International Civil Aviation Organization (ICAO) defines a UAS as *“An aircraft and its associated elements which are operated with no pilot on board”* [54]. There are two main categories of UAS: Remotely-Piloted Aircraft Systems (RPAS) and Unmanned Aerial Vehicles (UAVs). The former include *“A set of configurable elements consisting of a remotely-piloted aircraft, its associated remote pilot station(s), the required command and control links and any other system elements as maybe required, at any point during flight operation”* [54]. The latter is defined as *“A streamlined, flight ready machine that can take a flight without the help of a human”* [55,56].

In addition to being piloted or not, there is a wide variety of UAS that may be classified according to different criteria, e.g., their physical structure (fixed wings, multirotors or quadcopters); their weight (less than 2 kg, less than 25 kg or more than 25 kg); according to their topology (quadcopter, hexacopter, octocopter, etc.); the controlling method (autonomous, monitored, supervised, preprogrammed or remotely-controlled (R/C)); or other characteristics related to the components or devices integrated with the UAS.

Usually, a UAS is composed of a set of intrinsic components and a set of additional (external) devices that may complement the internal ones, adding more functionalities. Concretely, in order to consider an aerial vehicle as a UAS, it should contain the next intrinsic elements: flight controller, frame, engines, airscrews, ESCs (Electronic Speed Control), battery and emitter and receptor. The external components are, among others: GNSS (Global Navigation Satellite System), imaging/ranging sensors, camera stabilizer or gimbal, FPV (First Person View), ground control station, sensors and actuators.

All these internal and external components determine the functionalities and features that the UAS provides so that the more components the UAS relies on, the more features it usually provides.

## 4. Necessities for a Sustainable UAS Architecture

With the aim of evaluating whether the UAS existing on the market are prepared to be used in different application fields, we have conducted a study where we have identified the main features that must be provided by them. Then, based on these features, we have evaluated whether the most frequently-used UAS provide these features or not. This section provides an overview of this study and describes the main contributions derived from it, namely a new taxonomy of UAS features and the identification of the necessity for a sustainable UAS architecture.

**Figure 1:**
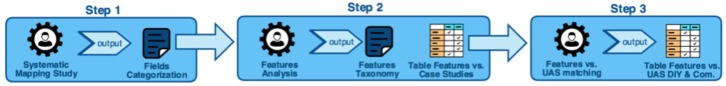
Process for identifying sustainable UAS necessity in a nutshell.

The steps that were performed for identifying the need for a sustainable UAS are listed below and outlined in Figure 1. For each step, the output is also specified:

- Step 1. Systematic Mapping Study (SMS): A systematic mapping study is carried out in order to identify a collection of representative case studies and areas where UAS are being used.
  – Output: Fields’ categorization: As a result of this step, the case studies are classified according to a particular categorization.
- Step 2. Feature analysis: A systematic analysis of the features required in each case is performed.
  – Outputs:
    * Features taxonomy: A new taxonomy where each feature is deeply defined and detailed. It represents the whole set of features that are present in all the case studies.
    * Features vs. case studies matching table: A table where the features that are required in each case study are summarized (grouped into the different categories).
- Step 3. Features vs. UAS matching: Based on an analysis of the UAS used in each case study and those that are more frequently commercialized, in this step, we compare the features identified in the case studies with those provided by the UAS in order to check whether the features may be provided or not by the UAS.
  – Output: The final result of this process is a table where we can easily check the features provided by all the UAS analyzed (both those used in the case studies and other commercial ones).

### 4.1. Features Required in Case Studies

In order to identify the features that are required in UAS systems, we performed an SMS of the literature where we analyzed the application fields and case studies where UAS were being used. The case studies evaluated were extracted not only from the academic domain, but also from commercial and industrial applications. Moreover, most of the projects studied were related to areas considered as critical for society [38,57], e.g., security, agriculture or water management.

In order to conduct the SMS, we formulated a set of research questions that we wanted to evaluate. These research questions are presented in Table 1.

To answer these research questions, the SMS has been carried out by querying well-known sources, such as Scopus (Scopus: https://www.scopus.com), IEEE Xplore (IEEE Xplore: http://ieeexplore.ieee.org/), ACM (ACM: http://dl.acm.org/), Elsevier (Elsevier: https://www.elsevier.es) and Springer (Springer: http://www.springer.com/), but also the latest editions of important conferences related to the topic, like International Conference on Unmanned Aircraft Systems (ICUAS 2013, 2014, 2015 and 2016), IEEE Aerospace Conference (AeroConf 2012, 2013, 2014, 2015 and 2016) and International Conference Actual Problems of Unmanned Aerial Vehicles Developments (APUAVD 2015).

Next, we show the search queries that we used in all the digital libraries. These queries were combined by using AND operators so that different composed queries were obtained:

1. (UAS OR drone OR UAV OR RPA OR “unmanned aerial vehicle” OR “unmanned aerial system” OR “remotely piloted aircraft”) (RQ1)
2. (“Case study” OR empirical OR experiment) (RQ2)
3. (Feature OR property OR characteristic) (RQ3)
4. (“Software engineering” OR algorithm OR method OR framework OR technology OR tool OR architecture OR system) (RQ1, RQ2 and RQ3)

As the first result of this search, 579 different papers were obtained. Based on the first analysis of their abstracts, their main goals and according to the relation with the aim of the study, 59 works were finally selected. After reading and analyzing all these works, we discarded 22 due to being out of the scope of the study (i.e., UAS were not used with a specific purpose, but just to test other research topics) and selected 37 works that were finally deeply analyzed.

Once the case studies selected were analyzed, we classified them according to the fields in which they were used (output of Step 1). The categories and sub-categories identified (including the works in each one) are described as follows:

A. Disasters and emergency: This refers to the occurrence of a fateful event that alters the usual behavior of the environment. The main activities related to this category are:
  A.1 Recognition and evaluation of damage in areas that suffered a natural disaster [58,59,60,61,62].
  A.2 Rescue in areas with difficult access [63,64,65,66].
B. Agriculture and cattle raising: activities that are performed to grow crops or raise animals with the aim of obtaining either products to be consumed by humans and other animals or raw materials for industry. The activities included in this category are:
  B.1 Precision agriculture [67,68,69,70,71,72].
  B.2 Shepherding activities [73,74,75].
C. Environmental control: tasks related to the inspection, surveillance and techniques applied to decrease or avoid any type of damage to the environment, in general, or to a specific ecosystem. Some examples are:
  C.1 Forest control and inventory [76,77,78,79].
  C.2 Air and atmosphere quality measurements [23,80,81].
  C.3 Geology and studies of places with difficult access [82,83].
  C.4 Bird nests control and study [84].
D. Audiovisual and entertainment: These refer to activities related to the integration of audio and visual techniques to produce audiovisual products (montages, recordings, films, etc.):
  D.1 Event recording or film production [80,85,86].
E. Surveillance and security: activities related to the integration of audio and visual techniques to produce audiovisual products (montages, recordings, films, etc.):
  E.1 Monitoring of structures and buildings [28,30,39,87].
  E.2 Surveillance in urban environments [21,88].

**Figure 2:**
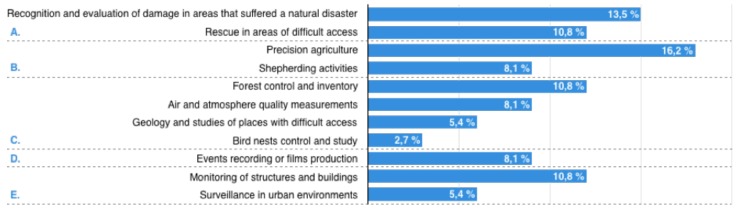
Categories identified and the number of case studies.

The subcategories identified and the number of case studies included in each one are graphically summarized in Figure 2.

The next step in the process consists of a deep analysis in order to identify the main features required in each case study (Step 2 in the process). The analysis has been performed by following the next process: (1) each case study was selected; (2) a list of potential features was identified based on the usage of the UAS in the case study; (3) the list of candidate features was reviewed by an expert committee, composed of a certified pilot and a group of UAS application developers; (4) the final list of features for the case study was provided. As an example, when the UAS must detect and avoid obstacles, processing and reasoning capacities are required. However, it is worth mentioning that some of the features may be provided by the use of different devices, e.g., processing capacity may be provided either by the UAS itself or by an external server that performs this computation. The utilization of a particular device to implement the feature may have advantages or limitations that should be considered when choosing a UAS for the case study.

Once the features have been identified, they are classified and formally specified by defining a taxonomy (output of Step 2). In particular, the features have been classified into four different main categories: (i) storage capacities; (ii) processing capacities; (iii) communication issues; (iv) and flexibility for configuration of both, hardware and software. Obviously, other categories and classifications could be possible. However, this taxonomy ensures that all the features identified are covered since it was driven by the analysis of the case studies and the identification of the features needed to face their challenges. Table 2 shows an excerpt of the taxonomy with the different features.

Based on the feature taxonomy and the SMS performed, we show a summary of the matching among the features and the case studies analyzed (output of Step 2). Concretely, Table 3 shows the features that are mandatory for each category (green tick), those that are not required (red cross) and, finally, those that would be recommended, but not mandatory (orange line), e.g., because the functionality may be achieved by a combination of other ones. Note that the features required for a category were obtained as the union of those required by each case study included in the category, considering that the feature may be provided by the UAS or an external component (this fact is not relevant for building this table).

### 4.2. Features Provided by UAS

According to the biggest commercial UAS distributor in the world [89,90], the best-seller and most used ones are: DJI S800 EVO (DJI S800 EVO: http://www.dji.com/es/product/spreading-wings-s800-evo/feature); DJI Phantom 3 (DJI Phantom 3: http://www.dji.com/es/products/phantom-3); DJI Phantom 4 (DJI Phantom 4: http://store.dji.com/product/phantom-4); TBS Discovery (TBS Discovery: http://www.team-blacksheep.com/products/product:98); Parrot Bebop (Parrot Bebop: https://www.parrot.com/us/es/drones/parrot-bebop-2); GHOST Drone Aerial 2.0 (GHOST Drone Aerial 2.0: http://www.ehang.com/); AirDog Drone (AirDog Drone: https://www.airdog.com); Hemav Drone (Hemav Drone: https://hemav.com/en); 3DR Solo Drone Quadcopter (3DR Solo Drone: https://3dr.com/solo-drone); Walkera Tali H500 (Walkera Tali H500: http://walkera-rc.es/Tali-H500.html); Yunnec Q500 (Yunnec Q500: https://www.yuneec.com/esES/drones-con-camara/typhoon-4k/vista-general.html); Intelligenia Dynamics Drone (Intelligenia Dynamics Drone: http://www.iuavs.com). However, most of these commercial UAS rely on a proprietary and closed architecture that is difficult and expensive to extend, and this causes the functionalities offered by the device to be limited. This problem has been usually faced by building Do-It-Yourself (DIY) UAS that provide a more flexible hardware architecture and may be built according to the necessities of the particular project. However, although providing more features, both their hardware architecture flexibility and the functionalities offered are still limited, so that they may not be used with different purposes.

In this context, this section focuses on the process (Step 3 in Figure 1) of analyzing which features (of the previously defined in the taxonomy) are provided by both: (i) the DIY UAS used in the case studies analyzed in the SMS and (ii) the set of UAS more frequently sold and used, according to [89,90]. The process to identify the features provided by each UAS was similar to the one performed for the case studies: (1) a UAS is selected, (2) the internal and external components that are integrated with it are enumerated; (3) the features provided by each component are identified; (4) the features provided by the UAS are defined, based on the union of the features provided by its components.

The matching between features and DIY UAS is presented in Table 4. In this case, the orange line indicates that the UAS partially provides the corresponding feature. That means that the UAS provides some of the functionalities that may be achieved by the feature, but not all of them, e.g., some UAS provide unidirectional communication instead of bidirectional, and thus, it may either receive orders or send information from/to the base station, but not both of them.

Table 4 provides interesting information regarding some features required by the case studies that were not provided by the UAS. As an example, none of the UAS used in the disasters and emergency category (A) provides processing features, although these features are required for the category (see Table 3). These features are replaced, in most cases, by storage capacity so that the images recorded are stored in the device and processed by the server later on, once the UAS has landed (e.g., in [60,64] or [65]).

In the case of commercial UAS, the matching between commercial UAS and features is presented in Table 5. Based on the analysis of the features provided by the commercial UAS, we extracted interesting conclusions, e.g., we realized that context sensitivity is only partially provided by DJI Phantom 4. Concretely, it relies on a set of ultrasonic sensors that, together with basic reasoning capabilities, avoid crashing into obstacles.

Based on the observation of both tables, we confirmed that none of the UAS (neither DIY, nor commercial) provides the whole set of features defined in our taxonomy, some of them being mandatory to use the device in each application field. This conclusion supports our claim for the necessity of a sustainable UAS that may be used for different purposes.

## 5. Our Approach: A General Multipurpose UAS Architecture

**Figure 3:**
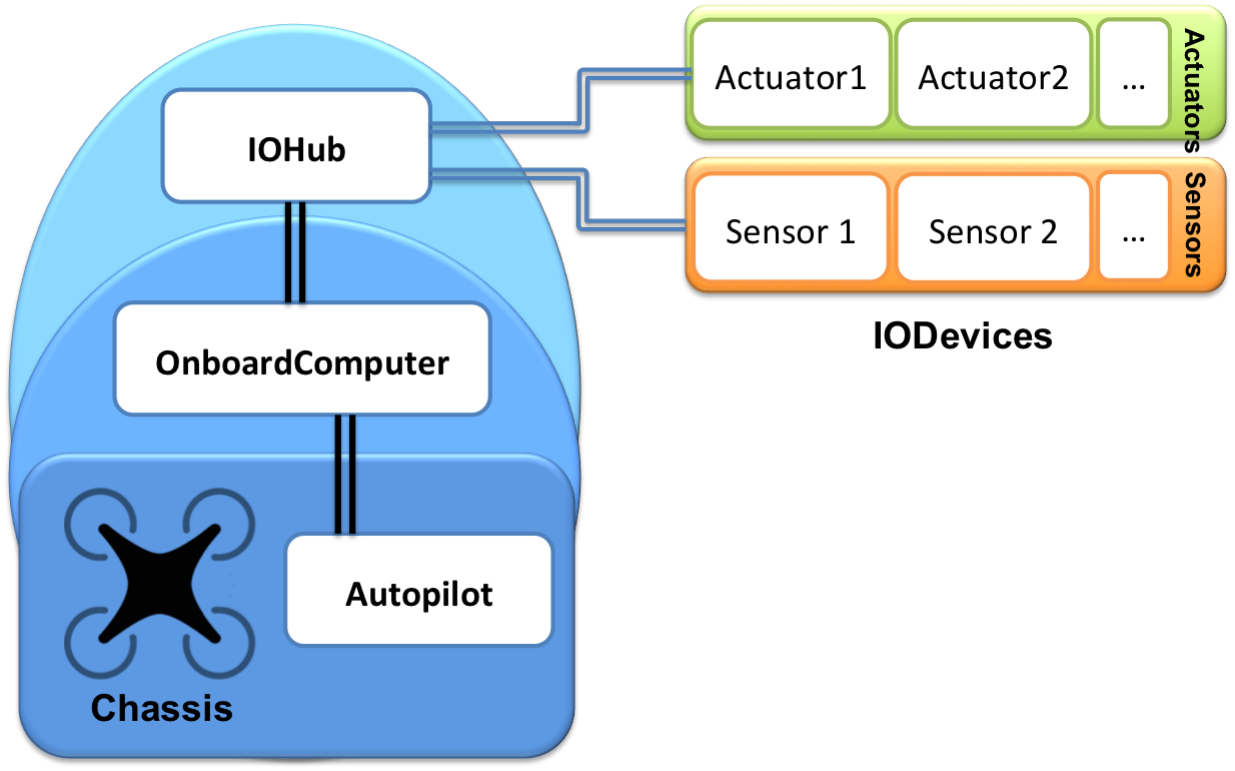
General architecture.

Based on the need for a multipurpose UAS architecture previously identified, this section presents a first approach to this open architecture. Our claim is that, based on this architecture, a UAS could be built to be used in any of the fields identified in Section 3 since it would provide all the features required in all these fields. The architecture proposed here takes as input the core of a UAS composed by the chassis and an AutoPilot. However, as has been previously presented, this core architecture (that all the UAS mentioned in Section 4 contain) lacks some functionalities that are necessary for the fulfillment of all the features identified in this work. Thus, the core architecture is also extended with additional layers that contain components that provide these features. Figure 3 shows a representation of the architecture proposed here.

As may be observed in Figure 3, the architecture is mainly based on the combination of three main components: AutoPilot, OnBoardComputer and IOHub. Next, we provide a deeper explanation of these three components and the features covered by them (which are also summarized in Table 6).

### 5.1. AutoPilot

The AutoPilot is the device responsible for receiving the information from the internal sensors of the UAS (e.g., flight altitude, GPS location, inclination, etc.) and sending the corresponding orders to the ESC (Electronic Speed Control) that controls the speed of the different rotors and, thus, the movement of the airscrews. In other words, this component stabilizes the UAS and keeps it flying. Moreover, the AutoPilot implements the communication protocol between the PC and UAS (PC-Drone) and allows the remote control (Remote-Drone). Finally, it allows programming easy tasks related to the route, such as going to a coordinate (e.g., “GoTo (x,y,z)”) or establishing flying restrictions (e.g., “AltitudeLimit = 80 feet”), or connecting to other existing route planning software in order to create a more complex planning (Programming and Route Planning Software). Note that the AutoPilot is integrated into the UAS so that these features are intrinsically provided by the UAS.

### 5.2. OnBoardComputer

Those UAS that only rely on the basic infrastructure (chassis, AutoPilot and basic electronic devices like the ESC) may just perform simple tasks, which cannot be modified on the fly (apart from basic actions like Return to Home). The OnBoardComputer provides the UAS with more computational capacity so that it may be able to carry out more complex actions (extensibility), e.g., actions based on processing images in real time during the flight.

The OnBoardComputer is usually based on an embedded operating system that enables the developer to program advanced actions on the UAS by using high level programming languages such as C or Python. Thus, by using the OnBoardComputer, the UAS may provide the next features: (i) adaptability, since the behavior of the UAS may be re-programmed both statically (before flying) and dynamically (during the fly); (ii) processing capacity, due to the complex calculations that may be performed on the fly in order to adapt the UAS behavior; (iii) reasoning, that is the natural consequence of being able to process data on the fly; (iv) storage capacity, since the data being processed by the OnBoardComputer must usually be stored.

### 5.3. IOHub

The IOHub controller (or micro-controller) allows the connectivity with external sensors and actuators so that the functionalities offered by the UAS may also be extended, providing new features that are not covered by the two previous components. In particular, on the one hand, the IOHub will receive the measurements taken by the sensors and pre-process them (e.g., by filtering or combining) before sending them to the OnBoardComputer; on the other hand, it will send the control signals from the OnBoardComputer to the actuators. Based on these actions, the IOHub is responsible for providing the next features: (i) external data acquisition/provision, since the sensors connected provide information about external magnitudes; (ii) context sensitive, again due to the awareness of the environment offered by the sensors connected; (iii) extensibility, this feature is not only provided because of hardware extensions, but also from the software perspective since the IOHub frees the OnBoardComputer from performing some monitoring and controlling tasks.

There is a wide range of sensors and actuators on the market that may be connected to the IOHub such as ultrasonic or infrared sensors to measure distances; weather sensors to assess temperature and other atmospheric magnitudes; light sensors to measure lumens intensity; light and acoustic actuators to react to an action; servomotors to, for instance, control a gimbal (allows holding and moving a camera). The important point with this architecture is that the user does not have to deal with the code needed for integrating these external components. This is done through automatic code generators that are based on a DSL (Domain Specific Language). Although it is out of the scope of this paper, next we give an overview of what the features that are the DSL covers just to better understand the context of our contribution.

### 5.4. DSL

A DSL has been developed that allows the user to perform the following tasks:

- Specify the devices that will compose the hardware architecture (image/ranging sensors, actuators, and so on). An initial catalog of devices is included within the DSL (e.g., GoPro Hero 3, Asus Xtion Pro Live or HC-SR04, just to cite a few)
- Based on this specification, the DSL also allows one to include restrictions, such as maximum weight, distance, etc.
- It is also possible to check that the type of connections among devices are correct.
- Once a hardware implementation has been defined, code generators automatically generate the skeleton of the code that is embedded on those devices.
- The DSL also allows one to program the flight plan and the actions to be carried out by the UAS, generating also the necessary code for each of the devices.
- Finally, the DSL generates the necessary documentation to comply with the process of registration of operations indicated by the law of the country where the work will be carried out (a few countries have been initially considered just to validate the proposal).

## 6. Implementation: An Instance of the Architecture

This section describes how the open architecture previously explained may be instantiated. In other words, it shows how a UAS based on this architecture may be designed and assembled. Additionally, the selection of the components for each part of the architecture has been driven by a review of the market where the existing alternatives for each component have been analyzed with the aim of selecting the best one in each case.

**Figure 4:**
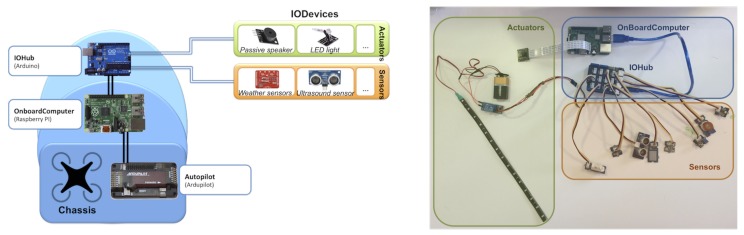
Implementation.

Figure 4 shows the concrete instance of the architecture with the selected devices for each part. These devices used for building the UAS are explained in the next subsections.

### 6.1. Chassis

The frame DJI F-450 with 750W rotors was selected. This chassis was selected since it has no legs, and this eases the tests and calibrations (the UAS did not take off during the tests). After being calibrated, we added the legs to the chassis so that it could fly.

### 6.2. AutoPilot

With the aim of selecting the best alternatives to be used in the architecture, we performed a comparison of a wide set of AutoPilots existing on the market. Concretely, 49 AutoPilots were evaluated according to: physical characteristics (weight, size, etc.), processing capacities (CPU, storage, etc.), system specifications (e.g., operating system, programming IDEs, etc.), functionalities provided (programming libraries available, interface connection, waypoints navigation, etc.) and commercial issues (such as price, company, license, etc.). From the 49 AutoPilots initially evaluated, a subset of 14 was discarded because of either a lack of supporting documentation or being out-dated. Finally, from the total set of 35 AutoPilots, we selected three as the candidates to be used based on the next criteria: (i) they are open-source; (ii) their reduced price (less than 200$); (iii) they are the ones with the best documentation provided by the supplier; (iv) they are easy to program. The three candidates are presented in Table 7 where a summary of their features is provided.

From the three candidates presented in Table 7, we selected for the implementation Ardupilot APM 2.6 due to, in addition to the features previously mentioned, its processing and storing capacities; the predefined directives that are programmed in their own device (waypoints navigation, Auto-TakeOff and landing, etc.); and the possibility to program the device and modify its behavior on the fly.

### 6.3. OnBoardComputer

In our selection of the best OnBoardComputers, we studied 111 micro-computers that may be acquired on the market. The characteristics that were compared in this case are: physical features, computational specifications, I/O interfaces, audiovisual interfaces, operating system and commercial issues. A total of 55 micro-computers was dismissed based on the complexity of either acquiring them or accessing the specifications. Like for the AutoPilot, we selected the devices according to the next criteria: being open-source, having a reduced price, with the best documentation provided by the supplier and being easily programmed. Based on these criteria, the three candidates to be used were: (i) Raspberry Pi 3, with an optimal processing capability, options to extend its storage and wireless connections; (ii) Raspberry Pi 2, similar to the previous one with the exception of the lack of wireless connections, but with the widest and most extended documentation; and (iii) ODROID XU-4, which provides better computing specifications than both Raspberry Pi, but for a higher price. Table 8 and Table 9 summarizes the features of these devices.

The candidate selected was Raspberry Pi 2 (Model B) (Raspberry Pi 2 (Model B): https://www.raspberrypi.org/products/raspberry-pi-2-model-b) with the operating system Raspbian (Raspbian: https://www.raspbian.org) (based on Linux). This device was chosen due namely to its high performance and reduced price, the great amount of documentation available online and the ease of connection with the AutoPilot (by means of USB and MAVLink (MAVLink Protocol: http://qgroundcontrol.org/mavlink/start) protocol). Note that although this component does not provide wireless connections, we only needed the connection with the AutoPilot, and this is why we did not use the Raspberry Pi 3 model.

### 6.4. IOHub

For the IOHub device, we studied a set of 76 commercial microcontrollers. In the first review, we dismissed 10 of them because of being out-dated or a lack of support by the supplier. In the second, and deeper review, another 42 were discarded due to the complexity of accessing their specifications or the supporting documentation. Finally, the three candidates selected were (see Table 10): (i) Arduino UNO, based on its high capacity, its reduced price and its ease of use; (ii) Arduino MEGA 2560, similar to the previous one, but with improved EEPROM, SRAM and Flash memories and more connections; and (iii) Arduino MKR1000, with similar characteristics to the previous ones and WiFi connection and without EEPROM memory.

In this case, the selected one was Arduino UNO since, although the three candidates selected were similar in characteristics, the documentation available on the Internet for this device and its reduced price were considered key factors. Moreover, an ultrasonic sensor and an actuator were connected to the IOHub by means of a shield interface of the Arduino UNO. This shield interface was incorporated to the Arduino UNO to ease the physical connections among sensors and actuators (avoiding the need for soldering to connect the external devices).

### 6.5. Final Assembly

**Figure 5:**
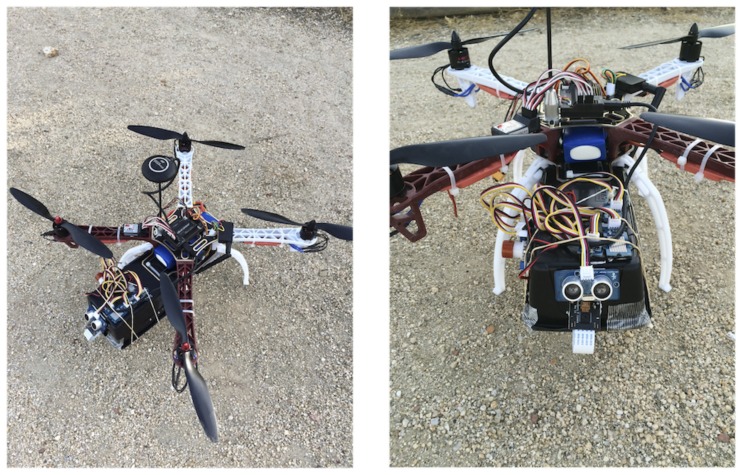
Photo of the UAS.

The final UAS built may be observed in Figure 5. It contains the selected components shown in Figure 4.

The software system that is embedded in the Raspberry Pi has been developed by using the DronKit API (DronKit API: http://python.dronekit.io/). This API allows the integration of all the installed devices and eases the communications by using the MAVLink protocol through a serial connection with a speed transmission of 57,600 bps (baud rate).

Finally, after assembling all the components and programming the software, a calibration process was performed since the incorporation of the external devices modifies the weight and gravity center of the UAS.

Based on the whole UAS built and the functionalities provided by it, we finally analyzed whether the device provided the features identified in this work, corroborating that they all were covered by it. Table 11 describes each feature and the concrete component/functionality of the UAS built that provides it.

### 6.6. Validation

Once the UAS was calibrated, the next step was to validate the prototype built. Due to the impossibility of reproducing all the case studies shown in this paper, we carried out a simulation by connecting the UAS to the SITL simulator (SITL simulator: http://ardupilot.org/dev/docs/sitl-simulator-software-in-the-loop.html). SITL is a state machine that allows one to check the system behavior by verifying that the output of the system is right according to the different entries and external events. Since SITL does not provide a graphical interface, we connected it to APM Planner Software (APM Planner: http://ardupilot.org/planner2/) for behavioral visualization. This combination allowed us to create several scenarios to test the operation of the device in different situations. Concretely, we tested the suitability of the device in one scenario for each category of the application fields identified in this work. Note that “Hardware-In-the-Loop” (HIL) simulations have been performed so that the different scenarios have been tested over the UAS hardware ensuring, thus, that the simulation is as close as possible to the actual situation. An example of these simulations may be found in [91], where we modeled a scenario for the measurement of different physical magnitudes in the context of an airport (environmental control and surveillance categories).

## 7. Conclusions

This work has presented a multipurpose UAS architecture that may be used to build sustainable UAS. Concretely, the need for this architecture was firstly introduced based on a mapping study that shows the lack of this kind of architecture. Secondly, a concrete implementation of the general architecture has been presented in order to illustrate the applicability of the approach. To create this concrete implementation, a deep analysis of the available devices for each part of the architecture has been performed. Notice that the architecture provides different benefits in terms of sustainability. On the one hand, the cost of monitoring a city by means of sensors may be highly reduced since all these sensors may be incorporated into the UAS (economic dimension); on the other hand, the multipurpose architecture ensures that the UAS built may be adapted to the different domains where UAS are being currently used so that the technical dimension of sustainability is fostered.

Next steps in this work imply leaving the simulation environment and jumping to a real one. We are now in this process, and the first results obtained have been as good as those obtained in the simulation.

## Figures and Tables

**Table 1:** Research questions.

Research Question	Main Motivation
RQ1: In which contexts and areas are UAS being currently used?	To collect a set of case studies and areas where UAS are being used and the purpose of using them.
RQ2: What techniques and technologies are applied to use UAS in the different areas?	To know the technologies that either are applied to UAS or that UAS provide and their maturity level.
RQ3: Which features must the UAS provide in order to be used in each area?	To know the features that a UAS must have in order to be used in the different areas identified.

**Table 2:** Feature taxonomy.

**Storage**	**Storage capacity**	Capacity for recording and persistently storing data into an electronic device.
**Processing**	**Processing capacity**	Capacity for executing calculations, operations and algorithms.
	**Reasoning**	Capacity for processing the data acquired by the UAS and taking automatic decisions accordingly. This feature is strongly coupled with “Processing capacity” since it is required for achieving Reasoning.
	**Context sensitive**	Capacity for acquiring data from the environment and reacting according to these data in order to preserve the security of the device. This features is also strongly related with “Reasoning” and “Processing capacity”.
**Communication**	**Communication PC-UAS**	Capacity for communicating the UAS with a server or Ground Station based on a wireless connection, such as WiFi (for a short distance) or radio (for a large distance).
	**Communication Remote-UAS**	Capacity for communicating the UAS with a remote radio-control.
	**Communication to external entity**	Property that enables the communication between the UAS and an external entity in order to send information (measurements, controlling parameters, images, etc.) or receive data (e.g. accessing to a Web service, communicating with another aircraft, etc.).
**Configuration**	**Extensibility**	Capacity for adding new components (sensors and/or actuators, cameras, ...) or interchanging those that are previously installed.
	**Programming**	Property that allows the automation of directives or rules to be used in concrete situations. This programming capacity may be performed at a low abstraction level (adding machine code directly to the autopilot) or at a higher abstraction level (based on the usage of particular programs that translate the code into machine code).
	**Route planning software**	Capacity for programming the UAS through a PC or mobile device by specific software for route planning. This software may be closed to modifications (usually proprietary) or open to be extended with new directives or to adapt the existing ones.
	**Adaptability**	Property that allows modifying the programmed tasks during the flight (modifications on the fly).

**Table 3:** Use case categories and features included in them.

		Storage	Processing			Communication			Configuration			
									Hardware	Software		
		Storage capacity	Processing capacity	Reasoning	Context sensitive	Communication PC-UAS	Communication Remote-UAS	Communication to External entity	Extensibility	Programming	Route planning software	Adaptability
A	Disasters and emergency	X	✓	✓	✓	✓	-	✓	X	X	X	X
B	Agriculture and cattle raising	✓	✓	✓	✓	-	✓	X	X	-	✓	✓
C	Environmental control	✓	-	X	X	X	✓	✓	✓	✓	X	X
D	Audiovisual and entertainment	✓	X	X	X	-	✓	X	✓	X	X	✓
E	Surveillance and security	X	✓	-	-	-	✓	✓	X	X	X	✓

**Table 4:** Matching between features and DIY UAS.

		Storage	Processing			Communication			Configuration			
									Hardware	Software		
		Storage capacity	Processing capacity	Reasoning	Context sensitive	Communication PC-UAS	Communication Remote-UAS	Communication to External entity	Extensibility	Programming	Route planning software	Adaptability
A	[58]	X	X	X	X	-	X	✓	X	-	✓	X
	[59]	X	X	X	X	X	✓	✓	X	X	X	X
	[60]	✓	X	X	X	✓	X	X	X	X	X	✓
	[61]	X	X	X	X	✓	X	✓	X	X	X	X
	[62]	X	X	X	X	✓	X	✓	-	X	X	X
	[63]	X	X	X	X	-	X	✓	-	X	X	X
	[64]	✓	X	X	X	X	✓	X	X	X	X	X
	[65]	✓	X	X	X	X	✓	X	X	X	X	X
	[66]	X	X	X	X	✓	X	✓	X	X	X	
B	[73]	-	X	X	X	X	✓	X	X	X	X	X
	[74]	✓	X	X	X	-	X	X	X	X	✓	X
	[75]	✓	X	X	X	✓	-	✓	X	X	X	X
	[67]	✓	X	X	X	✓	X	X	X	X	✓	X
	[68]	✓	X	X	X	X	X	X	X	X	✓	X
	[69]	✓	X	X	X	X	X	✓	X	X	✓	X
	[70]	X	X	X	X	✓	X	✓	X	X	X	-
	[71]	✓	X	X	X	X	✓	X	X	X	X	X
	[72]	✓	X	X	X	X	✓	X	X	X	X	X
C	[76]	✓	X	X	X	X	✓	X	X	X	X	X
	[77]	✓	X	X	X	X	✓	X	X	X	✓	X
	[78]	✓	X	X	X	✓	X	X	X	X	✓	X
	[79]	✓	X	X	X	X	✓	X	-	X	X	X
	[84]	X	X	X	X	X	✓	✓	-	X	X	X
	[82]	✓	✓	-	X	✓	X	X	X	✓	X	X
	[83]	✓	X	X	X	✓	X	X	X	X	✓	X
	[80]	✓	X	X	X	✓	X	✓	X	X	✓	X
	[81]	X	X	X	X	✓	X	✓	X	X	✓	✓
	[23]	X	✓	-	X	✓	X	✓	X	X	✓	
D	[80]	✓	X	X	X	X	✓	X	-	X	X	X
	[85]	✓	X	X	X	X	✓	✓	X	X	X	X
	[86]	X	X	X	X	✓	✓	✓	-	X	X	✓
E	[21]	X	-	-	X	X	X	X	X		X	
	[87]	✓	X	X	X	X	✓	X	X	X	X	X
	[39]	✓	X	X	X	X	✓	X	X	X	X	X
	[30]	✓	X	X	X	X	✓	X	X	X	X	X
	[28]	✓	X	X	X	X	✓	X	-	X	X	X

**Table 5:** Matching between features and commercial UAS.

	Storage	Processing			Communication			Configuration			
								Hardware	Software		
	Storage capacity	Processing capacity	Reasoning	Context sensitive	Communication PC-UAS	Communication Remote-UAS	Communication to External entity	Extensibility	Programming	Route planning software	Adaptability
DJI S800 EVO	X	X	X	X	X	✓	X	✓	X	✓	X
DJI Phantom 3	✓	X	X	X	X	✓	X	X	X	✓	X
DJI Phantom 4	✓	-	-	-	X	✓	X	X	X	✓	X
TBS Discovery	-	X	X	X	✓	✓	X	-	X	✓	X
Parrot Beebop	X	X	X	X	X	-	X	X	X	✓	X
GHOST Drone Aerial 2.0	X	X	X	X	X	✓	X	X	X	X	X
AirDog Drone	✓	✓	✓	X	X	✓	X	X	X	-	X
Hemav Drone	✓	✓	X	X	✓	X	X	✓	✓	X	-
3DR Solo Drone Quadcopter	✓	X	X	X	X	✓	✓	X	X	X	X
Walkera Tali H500	X	X	X	X	X	✓	X	X	X	X	X
Yuneec Q500	X	X	X	X	X	✓	X	X	X	X	X
Intelligenia Dynamics Drone	✓	-	✓	X	✓	X	X	✓	-	X	X

**Table 6:** Features provided by each component.

			AutoPilots	OnBoardComputers	IOHubs
Storage		Storage capacity		✓	
Processing		Processing capacity Reasoning Context sensitive		✓ ✓	✓
Communication		Communication PC-UAS Communication Remote-UAS Communication to External entity	✓ ✓		✓
Configuration	Hardware Software	Extensibility Programming Route planning software Adaptability	✓* ✓ ✓	✓**	✓

* *Basic*; ** *Complex*.

**Table 7:** Autopilot candidates

		Autopilot APM 2.6	Pixhawk PX4	Paparazzi Lisa/M 2
		*Physical specifications*		
Size (mm)		70x45x15	82x50x16	60x34x10
Weight (g)		28	38	10.8
DC in (V)		3.3 - 5	4.5 - 5	3.3 - 5
Power consumption (mAh)		600	800	200
		*Computing specifications*		
CPU		Atmega 2560 (16 MHz)	Cortex M4F (168 MHz)	STM32 (84 MHz)
Memory		4 (MB)	256 KB	256 KB
Storage		16 MB	2 MB	64 KB
Storage expansion (MB)		No	Yes (micro-SD)	No
Communication range (km) [RC mudule depends] [minimum]		7	5	1.61 (Xbee XSC only)
		*System specifications*		
Operating System/Firmware		ArduCopter-APM-2.0	PX4 Pro Autopilot	GINA Autopilot
Based on		Arduino	Unix/Linux	ARM7
Open source and code		✓	✓	✓
Programming IDE		✓(Arduino IDE)	✓	X
Programming libraries		✓	✓	X
Programming languages		C / Python / Matlab	C / Python	C / Python / OCAML
Route planning software		✓ (ex: MisionPlanner)	✓ (ex: MisionPlanner)	✓ (GINA Ground Control Station)
Wireless configuration		Radio telemetry	Radio telemetry	X
Open source communication protocol		MAVLink	MAVLink	X
Interface connection		USB	micro-USB	micro-USB
Serial ports		✓	✓	✓
GPIO / I2C ports		✓	✓	✓
Other ports		✓	✓	✓
		*Autopilot functions*		
Waypoints navigation		✓	✓	✓
Auto-Take Off & landing	&	✓	✓	✓
Altitude hold		✓	✓	✓
Air speed hold		✓	✓	X
Multi-UAV support		X	X	X
In-flight route editing		✓	✓	X
		*Others*		
Price ($) without GPS		109	199	199
Company/Project		DIY Drones Team	3DR	Paparazzi UAV
Website		link	link	link
License		Open-Source	Open-Source	Open-Source

**Table 8:** OnBoardComputer candidates.

	Raspberry Pi 3	Raspberry Pi 2	ODROID-XU4
	*Physical specifications*		
Size (mm)	86x56x18	86x57x18	82x58x22
Weight (g)	59	45	60
DC in (V)	5	5	5
Power consumption (mAh) Power source	800 Micro-USB / GPIO header	800 Micro-USB / GPIO header	1.000 DC jack
	*Computing specifications*		
SoC (System on a Chip)	Broadcom BCM2837	Broadcom BCM2836	Samsung Exynos 5 Octa (5422)
Architecture	ARM Cortex-A53	ARM Cortex-A7	ARM Cortex-A7
Cores	4	4	8
Frecuency	1.2 GHz	900 MHz	1.4 GHz
GPU	Broadcom VideoCore IV	Broadcom VideoCore IV	ARM Mali-T628 (695 MHz)
Memory	1 GB	1 GB	2 GB
Type	LPDDR2	LPDDR2	DDR3L
	*I/O interfaces and ports*		
Storage on-board	X	X	X
Flash slots (storage expansion)	micro-SD	micro-SD	micro-SD
SATA	X	X	X
PCIe (Peripheral Component Interconnect Express)	X	X	X
USB 2.0	4	4	1
USB 3.0	X	X	2
USB Type (device)	undefined	undefined	OTG 3.0
Ethernet	✓(10/100)	✓(10/100)	✓(10/100/1000)
WiFi	✓(b/g/n)	X	X
GSM	X	X	X
Bluetooth	✓(4.1)	X	X
I2C (Inter-Integrated Circuit)	✓	✓	✓
SPI (Serial Peripheral Interface)	✓	✓	✓
GPIO	17	17	✓
Analog	X	X	ADC
Camera port/bus	✓	✓	X
Others	UART	UART	UART & RTC battery

**Table 9:** OnBoardComputer candidates cont.

	Raspberry Pi 3	Raspberry Pi 2	ODROID-XU4
	*Audiovisual interfaces*		
Mic. In	X	X	X
Audio out	X	X	X
HDMI	✓(1.4)	✓(1.4)	✓(1.4)
LVDS (Low-Voltage Differential Signaling)	X	X	X
Others	Composite video	X	X
	*Operating system*		
Operating system / Firmware	Windows 10 / GNU Linux (ex: Raspbian)	Windows 10 / GNU Linux (ex: Raspbian)	GNU Linux / Android
Open source and code	✓	✓	✓
Programming IDE / SDK	✓	✓	✓
Programming libraries	✓	✓	✓
Programming languages	C / C++ / Python / Perl / Ruby / etc.	C / C++ / Python / Perl / Ruby / etc.	C / C++ / Java / etc.
	*Others*		
Price ($)	45	35	74
Company/Project	Raspberry Pi Foundation	Raspberry Pi Foundation	Hardkernel
Website	link	link	link
License	GPL Open-Source	GPL Open-Source	GPL Open-Source

**Table 10:** Autopilot candidates.

	Arduino UNO	Arduino MEGA 2560	Arduino MKR1000
	*Physical specifications*		
Size (mm)	69x54x14	102x54x11	56x26x6
Weight (g)	25	37	10
DC In (V)	7 - 12	7 - 12	5
Power consumption (mAh)	42	17	49
Power source	DC jack	DC jack	Micro-USB
	*Computing specifications*		
CPU	ATmega328P (16 MHz)	ATmega2560 (16 MHz)	SAMD21 Cortex-M0+ (48 MHz)
EEPROM	1 KB	4 KB	X
SRAM	2 KB	8 KB	32 KB
Flash	32 KB	256 KB	256 KB
Storage expansion (MB)	X	X	X
Ethernet	X	X	X
WiFi	X	X	✓
USB	✓(Regular)	✓(Regular)	✓(Micro)
Analog IN	6	16	7
Analog OUT	0	0	1
Digital IN	14	54	8
Digital OUT	6	15	4
UART port	1	4	1
External interrupts	2	6	8
Others connections	X	X	✓
Display	X	X	X
	*System specifications*		
Operating System/Firmware	None	None	None
Open source and code	✓	✓	✓
Programming IDE	✓(Arduino IDE)	✓(Arduino IDE)	✓(Arduino IDE)
Programming libraries	✓	✓	✓
Programming languages	C / Processing / C# / Python / ArduBlock / etc.	C / Processing / C# / Python / ArduBlock / etc.	C / Processing / C# / Python / ArduBlock / etc.
	*Others*		
Price ($) without GPS	20	35	31
Company/Project	Arduino	Arduino	Arduino
Website	link	link	link
License	CC Atribution Share-Alike	CC Atribution Share-Alike	CC Atribution Share-Alike

**Table 11:** Features provided by the UAS built.

			AutoPilot (APM 2.6)	OnBoard Computer (Rasp. Pi 2)	IOHub (Arduino UNO)
Storage		Storage capacity		Up to 32 GB	
Processing		Processing capacity		512 MB	
		Reasoning		Programming capacity	
		Context sensitive			Different sensors
Communication		Communication PC-UAS	Telemetry		
		Communication Remote-UAS	Radio		
		Communication to External entity			GSM communications
Configuration	Hardware	Extensibility			Different Sensors
	Software	Programming	C or Python	Different languages	
		Route planning software	APM Planner		
		Adaptability	Different connections		

## Supplementary Materials

Supplementary File 1
