# Supplementary material for: Towards the Use of Unmanned Aerial Systems for Providing Sustainable Services in Smart Cities
Source: Sensors (Basel). 2017 Dec 27;18(1):64. doi: 10.3390/s18010064 (PMC5795605; doi:10.3390/s18010064)
Supplement: Supplementary File 1 [file sensors-18-00064-s001.pdf]

Table 2: Feature taxonomy.

**Storage**	Storage capacity	Capacity for recording and persistently storing data into an electronic device.
**Processing**	Processing capacity	Capacity for executing calculations, operations and algorithms.
	Reasoning	Capacity for processing the data acquired by the UAS and taking automatic decisions accordingly. This feature is strongly coupled with "Processing capacity" since it is required for achieving Reasoning.
	Context sensitive	Capacity for acquiring data from the environment and reacting according to these data in order to preserve the security of the device. This features is also strongly related with "Reasoning" and "Processing capacity".
**Communication**	Communication PC-UAS	Capacity for communicating the UAS with a server or Ground Station based on a wireless connection, such as WiFi (for a short distance) or radio (for a large distance).
	Communication Remote-UAS	Capacity for communicating the UAS with a remote radio-control.
	Communication to external entity	Property that enables the communication between the UAS and an external entity in order to send information (measurements, controlling parameters, images, etc.) or receive data (e.g. accessing to a Web service, communicating with another aircraft, etc.).
**Configuration**	Extensibility	Capacity for adding new components (sensors and/or actuators, cameras, …) or interchanging those that are previously installed.
	Programming	Property that allows the automation of directives or rules to be used in concrete situations. This programming capacity may be performed at a low abstraction level (adding machine code directly to the autopilot) or at a higher abstraction level (based on the usage of particular programs that translate the code into machine code).
	Route planning software	Capacity for programming the UAS through a PC or mobile device by specific software for route planning. This software may be closed to modifications (usually proprietary) or open to be extended with new directives or to adapt the existing ones.
	Adaptability	Property that allows modifying the programmed tasks during the flight (modifications on the fly).

Table 3: Use case categories and features included in them.

		**Storage**	**Processing**			**Communication**			**Configuration**			
									Hardware	Software		
		Storage capacity	Processing capacity	Reasoning	Context sensitive	Communication PC-UAS	Communication Remote-UAS	Communication to External entity	Extensibility	Programming	Route planning software	Adaptability
**A**	**Disasters and emergency**	✗	✓	✓	✓	✓	–	✓	✗	✗	✗	✗
**B**	**Agriculture and cattle raising**	✓	✓	✓	✓	–	✓	✗	✗	–	✓	✓
**C**	**Environmental control**	✓	–	✗	✗	✗	✓	✓	✓	✓	✗	✗
**D**	**Audiovisual and entertainment**	✓	✗	✗	✗	–	✓	✗	✓	✗	✗	✓
**E**	**Surveillance and security**	✗	✓	–	–	–	✓	✓	✗	✗	✗	✓

Table 4: Matching between features and DIY UAS.

		Storage	Processing			Communication			Configuration			
									Hardware	Software		
		Storage capacity	Processing capacity	Reasoning	Contex sensitive	Communication PC-UAS	Communication Remote-UAS	Communication to External entity	Extensibility	Programming	Route planning software	Adaptability
A	[58]	✗	✗	✗	✗	–	✗	✓	✗	–	✓	✗
	[59]	✗	✗	✗	✗	✗	✓	✓	✗	✗	✗	✗
	[60]	✓	✗	✗	✗	✓	✗	✗	✗	✗	✗	✓
	[61]	✗	✗	✗	✗	✓	✗	✓	✗	✗	✗	✗
	[62]	✗	✗	✗	✗	✓	✗	✓	–	✗	✗	✗
	[63]	✗	✗	✗	✗	–	✗	✓	–	✗	✗	✗
	[64]	✓	✗	✗	✗	✗	✓	✗	✗	✗	✗	✗
	[65]	✓	✗	✗	✗	✗	✓	✗	✗	✗	✗	✗
	[66]	✗	✗	✗	✗	✓	✗	✓	✗	✗	✗	✓
B	[73]	–	✗	✗	✗	✗	✓	✗	✗	✗	✗	✗
	[74]	✓	✗	✗	✗	–	✗	✗	✗	✗	✓	✗
	[75]	✓	✗	✗	✗	✓	–	✓	✗	✗	✗	✗
	[67]	✓	✗	✗	✗	✓	✗	✗	✗	✗	✓	✗
	[68]	✓	✗	✗	✗	✗	✗	✗	✗	✗	✓	✗
	[69]	✓	✗	✗	✗	✗	✗	✓	✗	✗	✓	✗
	[70]	✗	✗	✗	✗	✓	✗	✓	✗	✗	✗	–
	[71]	✓	✗	✗	✗	✗	✓	✗	✗	✗	✗	✗
	[72]	✓	✗	✗	✗	✗	✓	✗	✗	✗	✗	✗
C	[76]	✓	✗	✗	✗	✗	✓	✗	✗	✗	✗	✗
	[77]	✓	✗	✗	✗	✗	✓	✗	✗	✗	✓	✗
	[78]	✓	✗	✗	✗	✓	✗	✗	✗	✗	✓	✗
	[79]	✓	✗	✗	✗	✗	✓	✗	–	✗	✗	✗
	[84]	✗	✗	✗	✗	✗	✓	✓	–	✗	✗	✗
	[82]	✓	✓	–	✗	✓	✗	✗	✗	✓	✗	✗
	[83]	✓	✗	✗	✗	✓	✗	✗	✗	✗	✓	✗
	[80]	✓	✗	✗	✗	✓	✗	✓	✗	✗	✓	✗
	[81]	✗	✗	✗	✗	✓	✗	✓	✗	✗	✓	✓
	[23]	✗	✓	–	✗	✓	✗	✓	✗	✗	✓	✓
D	[80]	✓	✗	✗	✗	✗	✓	✗	–	✗	✗	✗
	[85]	✓	✗	✗	✗	✗	✓	✓	✗	✗	✗	✗
	[86]	✗	✗	✗	✗	✓	✓	✓	–	✗	✗	✓
E	[21]	✗	–	–	✗	✗	✗	✗	✗	✓	✗	✓
	[87]	✓	✗	✗	✗	✗	✓	✗	✗	✗	✗	✗
	[39]	✓	✗	✗	✗	✗	✓	✗	✗	✗	✗	✗
	[30]	✓	✗	✗	✗	✗	✓	✗	✗	✗	✗	✗
	[28]	✓	✗	✗	✗	✗	✓	✗	–	✗	✗	✗

Table 5: Matching between features and commercial UAS.

	Storage	Processing			Communication			Configuration			
								Hardware	Software		
	Storage capacity	Processing capacity	Reasoning	Context sensitive	Communication PC-UAS	Communication Remote-UAS	Communication to External entity	Extensibility	Programming	Route planning software	Adaptability
**Commercial Drones**											
DJI S800 EVO	✗	✗	✗	✗	✗	✓	✗	✓	✗	✓	✗
DJI Phantom 3	✓	✗	✗	✗	✗	✓	✗	✗	✗	✓	✗
DJI Phantom 4	✓	–	–	–	✗	✓	✗	✗	✗	✓	✗
TBS Discovery	–	✗	✗	✗	✓	✓	✗	–	✗	✓	✗
Parrot Beebop	✗	✗	✗	✗	✗	–	✗	✗	✗	✓	✗
GHOST Drone Aerial 2.0	✗	✗	✗	✗	✗	✓	✗	✗	✗	✗	✗
AirDog Drone	✓	✓	✓	✗	✗	✓	✗	✗	✗	–	✗
Hemav Drone	✓	✓	✗	✗	✓	✗	✗	✓	✓	✗	–
3DR Solo Drone Quadcopter	✓	✗	✗	✗	✗	✓	✓	✗	✗	✗	✗
Walkera Tali H500	✗	✗	✗	✗	✗	✓	✗	✗	✗	✗	✗
Yuneec Q500	✗	✗	✗	✗	✗	✓	✗	✗	✗	✗	✗
Intelligenia Dynamics Drone	✓	–	✓	✗	✓	✗	✗	✓	–	✗	✗

Table 6: Features provided by each component.

			Autopilots	OnBoard Computers	IOHubs
Storage		Storage capacity		■	
Processing		Processing capacity		■	
		Reasoning		■	
		Contex sensitive			■
Communication		Protocol (PC-Drone)	■		
		Control (Remote-Drone)	■		
		External data acquisition/provision			■
Configuration	Hardware	Extensibility			■
	Software	Programming	■		
		Route planning software	■		
		Adaptability		■	
